# Current Guideline of Chest Compression Depth for Children of All Ages May Be Too Deep for Younger Children

**DOI:** 10.1155/2019/7841759

**Published:** 2019-06-19

**Authors:** Jang Hee Lee, Sang Kuk Han, Ji Ung Na

**Affiliations:** Department of Emergency Medicine, Kangbuk Samsung Hospital, Sungkyunkwan University School of Medicine, Seoul, Republic of Korea

## Abstract

**Aim:**

To determine whether the chest compression depth of at least 1/3 of the Anteroposterior (AP) diameter of the chest and about 5 cm is appropriate for children of all age groups via chest computed tomography.

**Methods:**

The AP diameter of the chest, anterior chest wall diameter, and compressible diameter (Cd) were measured at the lower half of the sternum for patients aged 1-18 years using chest computed tomography. The mean ratio of 5 cm compression to the Cd of adult patients was used as the lower limit, and the mean ratio of 6 cm compression to the Cd of adult patients was used as the upper limit. Also, the depth of chest compression resulting in a residual depth <1 cm was considered to cause internal injury potentially. With the upper and lower limits, the compression ratios to the Cd were compared when compressions were performed at a depth of 1/3 the AP diameter of the chest and 5 cm for patients aged 1-18 years.

**Results:**

Among children aged 1-7 years, compressing 5 cm was deeper than 1/3 the AP diameter. Also, among children aged 1-5 years, 5 cm did not leave a residual depth of 1 cm, potentially causing intrathoracic injury.

**Conclusion:**

Current pediatric resuscitation guidelines of chest compression depth for children were too deep for younger children aged 1-7 years.

## 1. Introduction

Clinical studies on adult cardiac arrest showed that at least 5 cm of chest compression depth improved recovery of spontaneous circulation and provided good survival rate and neurologic outcomes [1, 2]. On the other hand, a chest compression depth of 6 cm or more caused more frequent iatrogenic injury [3]. Based on these findings, the chest compression depth strongly recommended in the 2015 American Heart Association and European Resuscitation Council guidelines for adult CPR is at least 5 cm (2 inches) and less than 6 cm (2.5 inches) [4–6].

However, since clinical studies of pediatric CPR cannot be actively performed as in adults, most of the evidence for chest compression depth presented in the guidelines relies on consensus from experts and data from body measurements through chest CT. Prior to the 2010 guidelines, chest compression for children could be performed up to 1/2 of the APd of the chest by expert opinion [7. 8]. However, in two studies involving children's body measurement through chest CT conducted in 2009, it was found that 1/2 of the APd was too deep as the chest compression depth for children [9.10]. As a result, the American Heart Association and European Resuscitation Council guidelines of pediatric CPR have suggested a chest compression depth of at least one-third of the APd of the chest and about 5 cm for all children from the age of one to the onset of puberty since 2010 [7–12].

One observational study of pediatric CPR showed that chest compression depth greater than 5 cm was associated with improvement in short-term outcomes [13]. However, children under the age of 8 years were only 8 (10%) among the subjects of this study. Therefore, this study may not provide enough information for younger children. It is necessary to pay attention to the continuous change of the body shape since the preadolescent child is in the process of growing on. Older children and younger children may have substantial difference in body shape and therefore current guideline of chest compression may be too deep for younger children.

On the other hand, according to clinical studies in adult cardiac arrest, large proportions of inadequate chest compression depth than guidelines were reported [14, 15]. Mannequin studies for infant and pediatric CPR also reported shallower mean chest compression depth than guidelines [16, 17]. As a result, various attempts have been made to obtain the guidelines recommended compression depth. For example, using the novel two thumb technique in infants could create a deeper chest compression depth than the conventional chest compression method, which could have a positive effect on the hemodynamics [18–20]. However, it is also necessary to study whether the chest compression depth recommended in the guidelines is appropriate for children of all ages.

The purpose of this study was to determine whether the current guidelines suggesting chest compression depth of at least 1/3 of the APd of the chest and about 5 cm is appropriate for children of all age groups by body measurement using chest CT.

## 2. Materials and Methods

### 2.1. Study Design and Subjects

This retrospective observational study was conducted at a tertiary hospital in Seoul, Korea. From January 2006 to December 2018, patients aged 1 year and younger than 18 years who underwent chest CT scan for nontraumatic conditions were included. Exclusion criteria were severe anomalies of the chest or vertebrae, reflecting abnormal anatomical structures, or repeated chest CT scans within 1 year.

The study protocol was approved by the Institutional Review Board of Kangbuk Samsung Hospital (approval number 2018-07-028).

### 2.2. Sample Size Calculation

When Cohen's f^2^ is set to medium size of 0.15, the R^2^ is 0.13. And when the number of tested covariates is set to 5, alpha of 0.05, and power of 0.9, the estimated sample size for multiple linear regression was 116. When the age is between one year and 17 years, it is necessary to have more than 7 people by age. However, considering further subanalysis, we aimed to collect up to 30 subjects for each age group. For age groups for which we could not select 30 subjects, only the available subjects were selected. When there were more than 30 subjects, only 30 subjects were selected through randomization.

### 2.3. Data Collection and Analysis

Chest CT images of the finally selected subjects were analyzed by Picture Archiving and Communication Systems via prespecified methods. All chest CT images were taken using a Brilliance 64-slice CT Scanner (PHILIPS, Amsterdam, Nederland) with slice thickness of 1.5 mm, slice interval 1.5 mm, kVp of 100, mAs of 50, pitch of 0.798, 0.5 s rotation time, and 4×0.625 collimation during the study period. Image analysis was based on precontrast axial images. To delineate usual point of chest compression (middle of the lower half of the sternum), we first looked for the slice point number of the starting position of the manubrium (A) and the lower end of the sternal body (B). Based on these two slice point numbers, the lower 1/4 value was calculated as (A-B)/4 + B, and the closest slice point number was selected.

**Figure 1:**
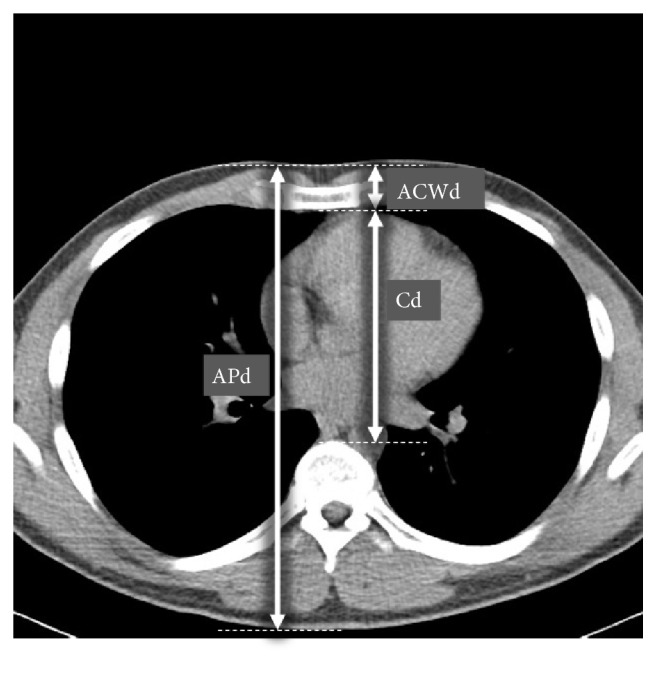
Measured diameters on an axial chest CT image at the level of the lower half of the sternum. APd was defined as the distance from the anterior skin to the posterior skin. ACWd was defined as the distance from the anterior skin to the posterior sternum. Cd was defined as the distance from the posterior sternum to the anterior vertebral body. APd: anteroposterior diameter; ACW: anterior chest wall diameter; C: compressible diameter.

Anteroposterior diameter (APd), the anterior chest wall diameter (ACWd), and the compressible diameter (Cd) were measured in selected axial image (Figure 1). APd was defined as the distance from the anterior skin to the posterior skin. ACWd was defined as the distance from the anterior skin to the posterior sternum. Cd was defined as the distance from the posterior sternum to the anterior vertebral body.

Image analysis was performed on a 27-inch 2560 × 1440 dpi resolution monitor and the length was measured using a 2D line measure tool embedded in the INFINITT PACS program (INFINITT Healthcare, Seoul, Korea).

Additional information such as patient's height and weight diagnosis was confirmed by Oder Communication System.

### 2.4. Outcomes

The mean values of the Cd for each age group were calculated. Then, the mean compression depth to Cd ratios when chest compressions were performed at 1/3 of the APd or 5 cm was compared and analyzed.

The chest compression depth suggested by the current guideline was evaluated on two criteria. First, the lower limit was set by mean compression depth to Cd ratio when 5cm compressions were delivered to adult, and the upper limit was set by mean compression depth to Cd ratio when 6 cm compressions were delivered to adult. In the unpublished pilot study, the mean compression depth of ratios to Cd when chest compressions were performed at 5 cm or 6 cm in adults was 47.3% and 56.7%, respectively. Second, the chest compression depth that created a residual depth of less than 1 cm was also set as the upper limit.

### 2.5. Statistical Analysis

All statistical analyses were performed using STATA 15.1 for Windows (StataCorp LLC, Texas, USA). Continuous data were presented as mean and standard deviation, and categorical data were expressed as n (%). Variables with normal distribution were compared using the* t*-test; the Mann-Whitney* U* test was used otherwise. In addition, linear regression analysis was performed to predict changes in Cd according to age and to compare. Statistical significance was considered at p <0.05.

## 3. Results

Of a total of 459 subjects, 17 were excluded (thoracic deformities: 9, overlapped study within 1 year: 8). Thus, a total of 442 subjects were included finally.

**Figure 2:**
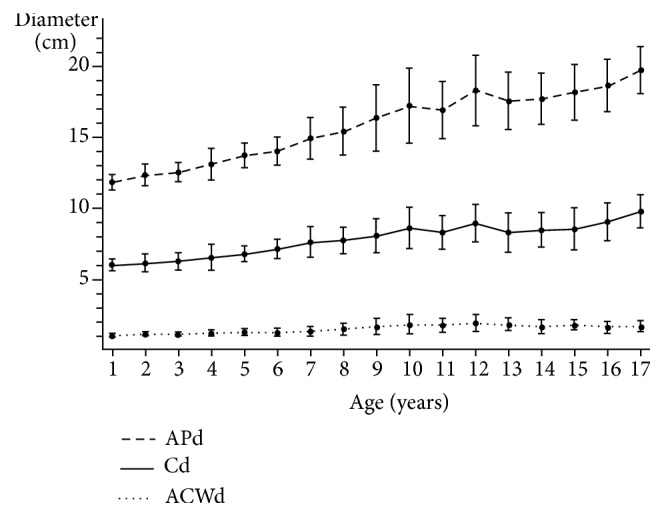
Means and standard deviation plots of APd, ACWd, and Cd by age group among patients aged 1–18 years. Black circles represent means and the error bars represent standard deviations. APd: anteroposterior diameter; ACWd: anterior chest wall diameter; Cd: compressible diameter.

The distributions of the number of subjects, male to female ratio, height, weight, and body mass index by age group are presented in Table 1. The distributions of APd, ACWd, Cd, and 1/3 of the APd by age group among patients younger than 18 years and older than 1 year are presented in Table 2. The means and standard deviation plots of APd, ACWd, and Cd by age group among patients younger than 18 years and older than 1 year are presented in Figure 2. Both Cd and APd showed linear relationship with age.

**Figure 3:**
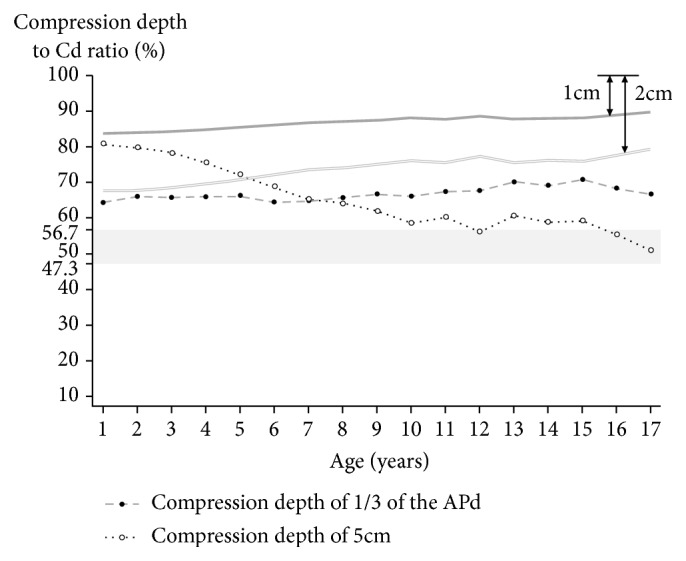
Compression depth to Cd ratios when chest compressions were performed at 1/3 of the APd or 5 cm by age group among patients aged 1–18 years. The white and black circles represent the mean ratios. The gray area represents the range of compression depth to Cd ratio when chest compressions were performed between 5 cm (47.3%) and 6 cm (56.7%) in adults. The gray bold line and the gray double line are the boundaries that indicate the residual depth of 1 cm and 2 cm, respectively. APd: anteroposterior diameter; Cd: compressible diameter.

Figure 3 shows the compression depth to Cd ratios when chest compressions were performed at 1/3 of the APd or 5cm. When compressed to 1/3 of the APd according to current pediatric CPR guidelines, compression depth to Cd ratios for all age groups was relatively higher than 56.7% which corresponds to 6 cm depth in adults. However, a residual depth of 1 cm or more could be achieved; even a 2 cm residual depth could be secured. The compression depth to Cd ratios, when chest compressions were performed at 5 cm, reaches about 80% by 1 year old and gradually decreases with age. The residual depth was less than 2 cm when the chest compressions were delivered by 5cm depth in children younger than 5 years old.

**Figure 4:**
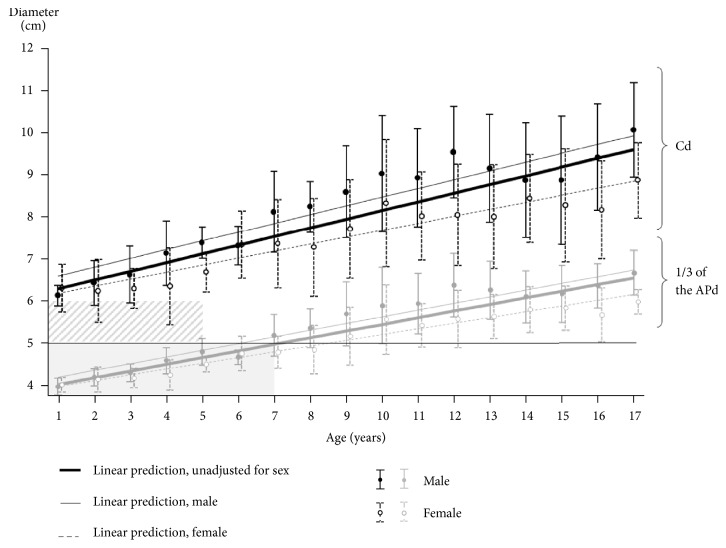
Linear regression analysis of Cd and 1/3 of the APd by age group among patients aged 1–18 years. Linear prediction lines (bold solid lines: unadjusted, fine solid and dotted lines: adjusted for sex) are shown with means (black circle) and standard deviations (error bars) by age. In some subjects with a Cd of less than 6 cm among 1 to 5 years of age, the residual depth is less than 1cm if chest compression is delivered by 5 cm depth (oblique-lined area). Chest compression depth is deeper when the chest was compressed by 5 cm than 1/3 of the APd in children between one to 7 years old (gray-colored area). APd: anteroposterior diameter; Cd: compressible diameter.

Multivariable linear regression analysis showed that the predicted compressible diameters were found to be 58.2 + 1.92 × age (years) + 7.44 × sex (male=1, female=0) mm (R2=0.47, p<0.01). The predicted one-third the AP diameters (mm) was found to be 37.29+ 1.51 × age (years) + 3.82 × sex (male=1, female=0) mm (R2=0.63, p<0.01).  Figure 4 shows an unadjusted regression line for Cd and 1/3 of the AP according to age and a sex-corrected regression line. A compression depth of 5 cm was deeper than 1/3 of the APd among patients older than 1 year and younger than 7 years (Figures 3 and 4). Also, among some patients between 1 and 5 years of age, a compression depth of 5 cm did not leave a residual depth of 1 cm (Figure 4).

The proportion of patients aged 1–5 years with a residual depth of less than 1 cm was more than 10%. The proportions among patients aged 1–2 years, 2–3 years, 3–4 years, and 4–5 years were 7/17 (41.2%), 4/14 (28.6%), 4/22 (18.2%), and 4/18 (22.2%), respectively. This proportion was less than 10% among patients aged 5–18 years.

## 4. Discussion

We evaluated whether the chest compression depth of at least 1/3 of the AP diameter of the chest and about 2 inches (5 cm) which is current guideline of pediatric CPR is appropriate for pediatrics of all age groups by body measurement using chest computed tomography.

Unlike previous studies, this study measured the actual compressible diameter by measuring the diameter of the anterior chest wall and AP diameter of vertebrae in all patients, without setting the remaining compressible diameter uniformly. In other words, individualization could be done to make more detailed body measurements.

In this study, the mean compression ratio to the actual compressible diameter was used as the reference value when chest compressions were performed at the depths of 5 cm and 6 cm in adults over 18 years old. When 5 cm and 6 cm of the chest compression depth were performed in adults over 18 years old, the mean compression ratios to the actual compressible diameter were 47.3 ± 8.3 (%) and 56.7 ± 9.9 (%). With the reference value, the compression ratio to the actual compressible diameter was compared when compressions were performed at the depth of 1/4 and 1/3 of the AP diameter of the chest in each age group under 18 years old. When compressed to 1/3 of the AP diameter of the chest which is current pediatric CPR guideline, compression ratios to the actual compressible diameter at all age groups under 18 years old were relatively higher than reference value, as the mean ratio was 67.0 ± 5.1 (%). When compressed to 1/4 of the AP diameter of the chest which is shallower than 1/3, compression ratio to the actual compressible diameter at all age groups under 18 years old was close to the reference value, as the mean ratio was 50.3 ± 3.8 (%). In addition, the compression ratio to the actual compressible diameter was compared with the reference value when compressing to 5 cm of the chest which is current pediatric CPR guideline in each age group under 18 years old. When compressed to 5cm of the chest, compression ratio to the actual compressible diameter under 15 years old was relatively higher than reference value. In the age group 15 years and older, compression ratio to the actual compressible diameter was close to the reference value. The compression ratio to the actual compressible diameter when compressed to 4 cm of chest was close to the reference value from 6 to 15 years of age. Under the age of 6 years, it was still deeper than the reference value even if 4 cm of the chest is compressed. Based on the results of this study, at least 1/3 of the AP diameter of the chest was too deep as the pediatric chest compression depth at all ages below 18 years of age, and at least 1/4 of the chest compression depth was appropriate. The chest compression of 5 cm was appropriate for aged 15 years and over, but it was found that the chest compression depth of 4 cm was appropriate for those under 15 years old. However, 4 cm was still deep under 6 years old.

This study has several limitations. First, this study was a retrospective, observational study based on body measurement using chest CT. Because it was not a clinical study, it was not verified whether the chest compression depth suggested in this study leads to the improvement of outcome for actual pediatric cardiac arrest patients. Second, the mean age of adults among whom reference values were determined was 62.5 ± 14.3 years, which was relatively high. However, the mean body mass index of the adults was 22.86 ± 4.18 kg/m^2^, which was within the normal range, so it would be reasonable to use it as the reference value for the body measurement. Third, because this study was conducted on patients who underwent chest CT at one university hospital in South Korea, they would not represent the whole race. Fourth, the study did not examine healthy patients because the chest CT scans were taken to examine diseases. Therefore, the subjects' bodies may be smaller than those of a healthy group. However, it is considered that the differences were insignificant because cases that did not show normal anatomical structure due to severe anomalies in the chest or vertebrae were excluded. Fifth, actual CPR is performed in the supine position with the arms lowered while the respirations are stopped, but the chest CT is taken with the arms raised in the inspiratory state. This may lead to a change in the position of the heart, which implies that the body measurements obtained using chest CT may differ from those observed during CPR [21].

## 5. Conclusion

According to the results of this study, the uniform guideline regarding chest compression depth for children of all ages was too deep for younger children. Particularly, among patients aged 1–7 years, a depth of 5 cm was deeper than 1/3 of the APd. Also, among patients aged 1–5 years, a compression depth of 5 cm was too deep and did not leave a residual depth of 1 cm, which could result in intrathoracic organ injury.

## Figures and Tables

**Table 1:** The distribution of number of subjects, male to female ratio, height, weight and BMI by age group among patients aged 1–18 years.

Age, years	n	Male n (%)	Female n (%)	Height, cm	Weight, kg	BMI, kg/m^2^
1	17	9 (52.9%)	8 (47.1%)	86.5 ± 11.7	11.8 ± 1.8	16.4 ± 3.1
2	14	7 (50.0 %)	7 (50.0%)	92.0 ± 5.8	13.6 ± 1.4	15.9 ± 1.5
3	22	11 (50.0 %)	11 (50.0%)	98.6 ± 4.2	14.9 ± 1.6	15.3 ± 1.4
4	18	9 (50.0 %)	9 (50.0%)	106.0 ± 5.6	17.9 ± 2.6	15.7 ± 1.5
5	24	10 (41.7 %)	14 (58.3%)	113.2 ± 5.2	20.5 ± 3.2	16.3 ± 1.8
6	30	12 (40.0 %)	18 (60.0%)	121.1 ± 4.2	22.8 ± 4.0	15.7 ± 2.1
7	26	15 (57.7 %)	11 (42.3%)	125.3 ± 5.5	25.0 ± 4.7	15.7 ± 1.7
8	26	17 (65.4 %)	9 (34.6%)	131.0 ± 6.8	29.5 ± 7.9	17.6 ± 3.7
9	30	21 (70.0 %)	9 (30.0%)	137.5 ± 6.4	35.7 ± 10.7	18.5 ± 3.7
10	28	18 (64.3 %)	10 (35.7%)	142.8 ± 6.9	38.6 ± 11.7	18.9 ± 4.6
11	29	14 (48.3 %)	15 (51.7%)	145.4 ± 9.5	39.9 ± 10.2	18.5 ± 3.0
12	30	21 (70.0 %)	9 (30.0%)	158.0 ± 7. 6	49.5 ± 12.6	19.8 ± 3.1
13	30	12 (40.0 %)	18 (60.0%)	158.9 ± 8.7	49.4 ± 9.5	19.2 ± 2.8
14	29	14 (48.3 %)	15 (51.7%)	163.8 ± 10.7	51.0 ± 9.5	19.0 ± 2.7
15	30	22 (73.3%)	8 (26.7%)	167.6 ± 10.1	53.9 ± 8.8	19.1 ± 2.2
16	30	25 (83.3%)	5 (16.7%)	171.9 ± 8.1	56.1 ± 8.6	18.9 ± 2.5
17	29	26 (89.7%)	3 (10.3%)	174.8 ± 7.7	61.1 ± 10.7	19.9 ± 3.0

Data were presented with means ± standard deviations

**Table 2:** APd, ACWd, Cd, and 1/3 of the AP diameter by age group among patients aged 1–18 years.

Age, years	n	APd, cm	ACWd, cm	Cd, cm	1/3 of the APd, cm
1	17	12.0 ± 0.6	1.1 ± 0.1	6.2 ± 0.4	4.0 ± 0.2
2	14	12.5 ± 0.7	1.2 ± 0.2	6.3 ± 0.6	4.2 ± 0.3
3	22	12.7 ± 0.7	1.2 ± 0.1	6.5 ± 0.6	4.2 ± 0.2
4	18	13.3 ± 1.1	1.3 ± 0.2	6.7 ± 0.9	4.4 ± 0.4
5	24	13.9 ± 0.8	1.3 ± 0.2	7.0 ± 0.6	4.6 ± 0.3
6	30	14.1 ± 1.0	1.3 ± 0.3	7.3 ± 0.7	4.7 ± 0.3
7	26	15.0 ± 1.5	1.4 ± 0.3	7.8 ± 1.1	5.0 ± 0.5
8	26	15.5 ± 1.7	1.6 ± 0.4	7.9 ± 0.9	5.2 ± 0.6
9	30	16.5 ± 2.3	1.7 ± 0.6	8.3 ± 1.2	5.5 ± 0.8
10	28	17.3 ± 2.6	1.9 ± 0.7	8.8 ± 1.4	5.8 ± 0.9
11	29	17.0 ± 2.0	1.8 ± 0.5	8.5 ± 1.2	5.7 ± 0.7
12	30	18.4 ± 2.5	2.0 ± 0.6	9.1 ± 1.3	6.1 ± 0.8
13	30	17.6 ± 2.0	1.9 ± 0.4	8.5 ± 1.4	5.9 ± 0.7
14	29	17.8 ± 1.8	1.7 ± 0.5	8.7 ± 1.2	5.9 ± 0.6
15	30	18.3 ± 2.0	1.8 ± 0.4	8.7 ± 1.5	6.1 ± 0.7
16	30	18.7 ± 1.8	1.7 ± 0.4	9.2 ± 1.3	6.2 ± 0.6
17	29	19.8 ± 1.6	1.7 ± 0.4	9.9 ± 1.2	6.6 ± 0.6

APd: anteroposterior diameter; ACWd: anterior chest wall diameter; Cd: compressible diameter.

## Data Availability

The data used to support the findings of this study are available from the corresponding author upon request.
